# Attenuated Acceleration to Leukemia after Ezh2 Loss in Nup98-HoxD13 (NHD13) Myelodysplastic Syndrome

**DOI:** 10.1097/HS9.0000000000000277

**Published:** 2019-07-22

**Authors:** Victoria Y. Ling, Jesslyn Saw, Cedric S. Tremblay, Stefan E. Sonderegger, Emma Toulmin, Jacqueline Boyle, Sung Kai Chiu, Steven W. Lane, Stephen B. Ting, David J. Curtis

**Affiliations:** 1 Australian Centre for Blood Diseases, Central Clinical School, Monash University, Melbourne, Australia; 2 QIMR Berghofer Medical Research Institute, Brisbane, Australia; 3 Royal Brisbane and Women's Hospital, Brisbane, Australia; 4 Department of Haematology, Eastern Health, Melbourne, Australia; 5 Department of Clinical Haematology, Alfred Health, Melbourne, Australia.

## Abstract

Supplemental Digital Content is available in the text

Myelodysplastic syndromes (MDS) are clonal hematopoietic stem cell disorders characterized by dysplastic blood cell morphology, ineffective hematopoiesis and a high rate of transformation to acute myeloid leukemia (AML).^1^ Epigenetic dysregulation underpins the pathogenesis of MDS, with recurrent mutations in epigenetic regulators including *TET2* (20%), *ASXL1* (14%), *DNMT3A* (12%), *EZH2* (6%) and *IDH1/2* (5%).^2,3^*EZH2*, or Enhancer of zeste homolog 2, is a histone methyltransferase and member of the highly conserved polycomb group of proteins, with important roles in regulating gene expression to coordinate self-renewal and differentiation of hematopoietic stem cells (HSCs).^4^*EZH2* loss-of-function mutations have an adverse effect on prognosis in MDS.^2^ Herein, we describe an *in vivo* model of attenuated acceleration to leukemia transformation with *Ezh2* deletion in a mouse model of MDS.

*EZH2*, together with other core subunits *EED*, *SUZ12,* and *RBBP4* form the polycomb repressor complex 2 (PRC2) complex responsible for the repressive tri-methylation modification of lysine 27 on histone 3 (H3K27me3).^5^*EZH1* and *2* are the only histone methyltransferases responsible for the H3K27 mark in mammals and functional redundancy exists with *EZH1*.^6^*EZH2* has important roles in maintaining HSC identity via repression of differentiation genes.^4^

*EZH2* mutations are usually loss-of-function in a broad range of myeloid malignancies including MDS, myeloproliferative neoplasms (MPNs) and AML.^7–9^ In MDS, mutations occur within *EZH2* in 6% of cases, however PRC2 is dysregulated in a larger subset of MDS (potentially 25–30% of cases) via gene deletion (del7q36.1) (3–4%),^3^*ASXL1* mutation (20%), which inhibits PRC2 function,^10^ or mutations in other PRC2 components (1–2%).^11,12^*EZH2* mutations occur rarely in *de novo* AML (∼2%),^9^ but are relatively enriched in AML arising from a precedent MDS (9%).^13^*EZH2* mutations are a poor prognostic indicator in MDS overall,^2^ including low-risk MDS, where *EZH2* mutation defines a subset with adverse clinical outcomes.^14^

In mouse models, loss of *Ezh2* leads to fatal defects in fetal hematopoiesis, although inducible loss in adult mice leads to a milder phenotype including retained self-renewal of HSCs that are able to engraft in secondary recipients.^6^ This may reflect increased dependency on *Ezh2* in highly proliferative fetal HSCs in the liver, compared to quiescent adult HSCs or may reflect *Ezh1* compensation.^15^ However, the complete loss of PRC2 activity in *Eed* knockout mice, leads to pancytopenia, defective differentiation and inability to compete with wild-type cells in competitive transplants, demonstrating integral roles of PRC2 signaling in hematopoiesis.^16^ Inducible *Ezh2* knock-out mice develop hematological malignancies with MDS, MDS/MPN^17–19^ and T-acute lymphoblastic leukaemia (ALL)^20^ described, but after a long latency suggesting cooperating mutations are required for transformation. Correspondingly, *Ezh2* deletion combined with *Tet2* deletion^17^ or *Runx1* mutation^18^ accelerated the MDS disease seen in these respective mouse models.

The *Nup98:HoxD13* transgenic (*NHD13*^*T*^) mouse model of MDS and secondary leukemia recapitulates key phases of human disease including a cytopenic phase which progresses at variable latencies to acute leukemia between 6 and 14 months.^21^*NHD13*^*T*^ mice express, under the control of the hematopoietic-specific *Vav* promoter, a fusion oncogene comprising the Nup98 nucleoporin protein and the homeobox protein HoxD13. The *Nup98:HoxD13* fusion is found rarely in human MDS or AML, however leukemia arising in *NHD13*^*T*^ is driven by the upregulation of Hox genes, a common mechanism in human disease.^22^ Additionally, epigenetic dysregulation appears to be an important contributor to Nup98-rearranged leukemia, as evidenced by its frequent fusions with epigenetic regulators.^22^ Given the driving role of *EZH2* dysregulation in MDS and its poor prognostic implications, we sought to study the effects of *Ezh2* loss-of-function in the *NHD13*^*T*^ mouse model. As Hox gene overexpression is also observed in *EZH2*-deleted MDS/AML,^23^ we hypothesized that the additive upregulation of Hox genes might provide a mechanism of cooperation between *Ezh2* deletion and *Nup98:HoxD13*. In long-term survival studies, we found that *Ezh2* deletion minimally accelerated leukemia development and death in *NHD13*^*T*^ demonstrating limited contribution to disease pathogenesis in the context studied.

*NHD13*^*T*^ mice were crossed with *Ezh2*^*fl/fl*^ mice^24^ expressing the polyinosinic:polycytidylic acid (poly (I:C))-inducible Mx1-cre recombinase (*Mx*^*T*^) to generate *NHD13*^*T*^ with *Ezh2* deletion (*NHD13*^*T*^*;Mx*^*T*^*;Ezh2*^*Δ/Δ*^) and control groups: wild-type (WT) (*Ezh2*^*fl/fl*^ or *Mx*^*T*^), *Ezh2*-deleted only (*Mx*^*T*^*;Ezh2*^*Δ/Δ*^) or *NHD13*^*T*^ only (*NHD13*^*T*^*;Ezh2*^*fl/fl*^ or *NHD13*^*T*^*;Mx*^*T*^). Poly (I:C) was administered intraperitoneally (6 injections over 2 weeks) to induce Cre recombinase and *Ezh2* deletion in 8 to 12-week-old mice. Mice were monitored by monthly peripheral blood analyses and welfare scoring and culled at disease onset (Fig. 1A).

**Figure 1:**
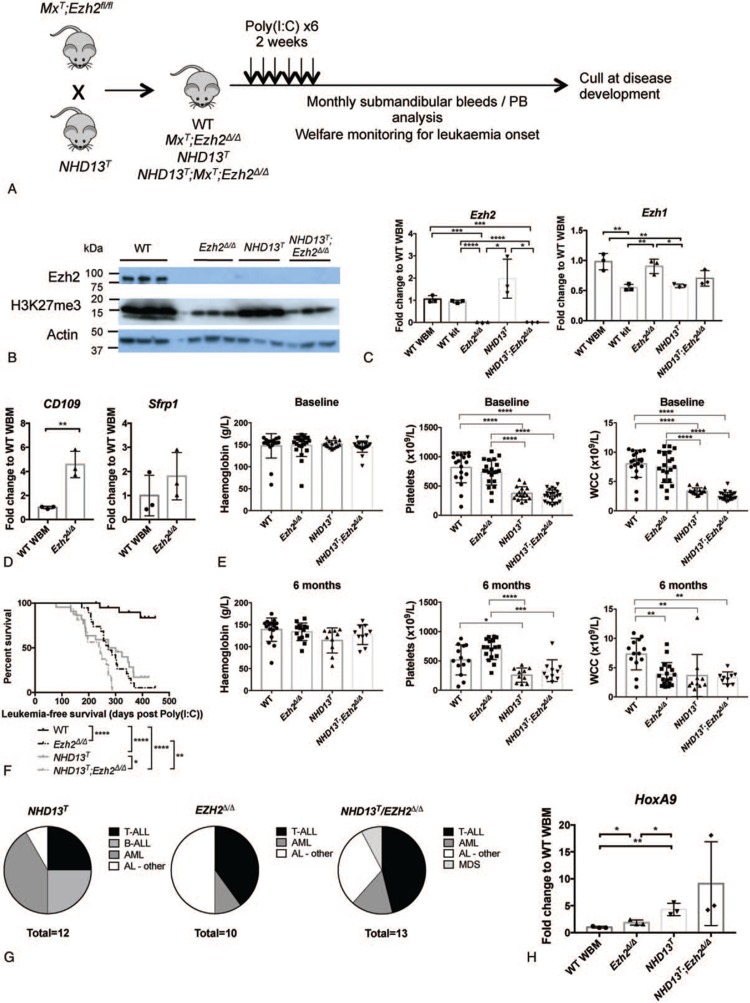
**Long-term effects of *Ezh2* deletion in *NHD13***^***T***^** leukemia.** A) Breeding and experimental schema for long-term survival assessment. PB, peripheral blood. B) Western blot demonstrating Ezh2 and H3K27me3 protein levels in *Ezh2*^*Δ/Δ*^, *NHD13*^*T*^ and *NHD13*^*T*^; *Ezh2*^*Δ/Δ*^ leukemias compared to kit-enriched wild-type bone marrow from adult C57BL/6J mice (WT), relative to an Actin loading control. Leukemic bone marrow samples of each genotype were taken from selected mice listed in Supplementary Table 1 (Supplemental Digital Content) and loaded in the listed order. C) *Ezh2* and *Ezh1* RNA expression levels as measured by qPCR in *Ezh2*^*Δ/Δ*^, *NHD13*^*T*^ and *NHD13*^*T*^; *Ezh2*^*Δ/Δ*^ leukemias compared to wild-type whole bone marrow (WT WBM) and WT kit-enriched bone marrow (WT kit). Samples were taken from the same mice as for B). D) Expression of Ezh2 targets as measured by qPCR. E) Peripheral blood counts prior to (baseline) and at 6 months post Poly (I:C). WCC, white cell counts. F) Kaplan-Meier survival curve after Poly (I:C) administration for each genotype. G) Proportions of disease types analyzed at time of cull for each genotype. ALL, acute lymphoblastic leukemia; AML, acute myeloid leukemia; MDS, myelodysplastic syndrome; AL, acute leukemia. H) *HoxA9* expression by qPCR in *Ezh2*^*Δ/Δ*^, *NHD13*^*T*^ and *NHD13*^*T*^; *Ezh2*^*Δ/Δ*^ leukemias compared to WT WBM. Statistics for qPCR experiments show results of unpaired *t* tests. Statistics for peripheral blood analyses show results of ANOVA testing. ^∗^*P* < 0.05, ^∗∗^*P* < 0.01, ^∗∗∗^*P* < 0.001 and ^∗∗∗∗^*P* < 0.0001.

We confirmed absence of *Ezh2* RNA and protein in bone marrow cells from *Ezh2*-deleted mice (*Mx*^*T*^*;Ezh2*^*Δ/Δ*^ and *NHD13*^*T*^*;Mx*^*T*^*;Ezh2*^*Δ/Δ*^) (Fig. 1B and C, Supplementary Table 1, Supplemental Digital Content). Consistent with this, H3K27me3 was reduced although not absent (Fig. 1B). Expression of *Ezh1* was maintained, although not upregulated, in *Mx*^*T*^*;Ezh2*^*Δ/Δ*^ and *NHD13*^*T*^*;Mx*^*T*^*;Ezh2*^*Δ/Δ*^ bone marrow and may explain the persistence of H3K27me3 (Fig. 1C). As H3K27me3 acts to repress gene expression, we confirmed gene de-repression consequences of *Ezh2* deletion in our model. In *Mx*^*T*^*;Ezh2*^*Δ/Δ*^, we observed increased *CD109*, a gene repressed by *Ezh2* with no *Ezh1* compensation (Fig. 1D).^19^ Conversely, there was no significant increase in *Sfrp1*, a gene regulated by both *Ezh2* and *Ezh1* (Fig. 1D).^19^ Maintained *Nup98:HoxD13* expression was confirmed in *NHD13*^*T*^ bone marrow by polymerase chain reaction (PCR) (Supplementary Figure 1, Supplemental Digital Content). Interestingly, bone marrow cells from *NHD13*^*T*^ mice had markedly reduced Ezh2 protein expression despite normal mRNA levels, which suggested a post-transcriptional down-regulation of Ezh2. Nevertheless, *NHD13*^*T*^ cells had normal levels of the H3K27me3 mark (Fig. 1B). *Ezh2* deletion in *NHD13*^*T*^ was able to reduce H3K27me3 to levels comparable with *Mx*^*T*^*;Ezh2*^*Δ/Δ*^ mice and thus still represented a suitable model in which to assess consequences of *Ezh2* deletion in a Hox-driven model of MDS.

EZH2 loss-of-function is associated with a poorer prognosis in MDS.^2^ We therefore sought to examine whether *NHD13*^*T*^*;Mx*^*T*^*;Ezh2*^*Δ/Δ*^ mice would have a shortened latency to leukemia development compared to the single mutation (*Mx*^*T*^*;Ezh2*^*Δ/Δ*^ or *NHD13*^*T*^) alone. *NHD13*^*T*^*;Mx*^*T*^*;Ezh2*^*Δ/Δ*^ mice had similar peripheral blood parameters to *NHD13*^*T*^ only mice, with leucopenia and thrombocytopenia by 3 months of age (baseline) and macrocytic anemia by 9 months post poly (I:C). *Mx*^*T*^*;Ezh2*^*Δ/Δ*^ mice had similar counts to WT at baseline but developed mild leucopenia by 6 months and anemia by 9 months after poly (I:C) (Fig. 1E).

*NHD13*^*T*^ and *Mx*^*T*^*;Ezh2*^*Δ/Δ*^ mice had shortened overall survivals compared with WT mice (median 287 days and 273 days, respectively vs undefined, p < 0.0001). Loss of *Ezh2* in *NHD13*^*T*^ mice conferred additional reduction in median survival (median 241 days, vs *NHD13*^*T*^, p = 0.010 and vs *Mx*^*T*^*;Ezh2*^*Δ/Δ*^, p = 0.0042), noting however, this was only 32 days shorter than with loss of *Ezh2* alone (Fig. 1F). At time of death, *Mx*^*T*^*;Ezh2*^*Δ/Δ*^, *NHD13*^*T*^ and *NHD13*^*T*^*;Mx*^*T*^*;Ezh2*^*Δ/Δ*^ mice developed a broad range of hematologic malignancies including T-ALL, AML, B-ALL, and MDS (Fig. 1G and Supplementary Tables 2, 3, and 4, Supplemental Digital Content). There was a significant proportion of other acute leukemias that expressed dual-lineage markers akin to mixed phenotype acute leukemias and those that did not express B/T/myeloid markers (acute leukemia (AL)-other). There were no significant differences in proportions of leukemic subtypes as determined by pairwise Fisher exact testing (Supplementary Table 5, Supplemental Digital Content). Analysis of hematopoietic stem and progenitor cell (HSPC) subpopulations from non-leukemic mice demonstrated an expansion of multipotent progenitor (MPP)3/4 cells (CD48^+^, CD150^−^ fraction of the lineage-negative, Sca1-positive, ckit-positive (LSK) population), which contain the granulocyte/macrophage (GM)-committed and lymphoid-committed MPPs,^25^ in *NHD13*^*T*^*;Mx*^*T*^*;Ezh2*^*Δ/Δ*^ and *Mx*^*T*^*;Ezh2*^*Δ/Δ*^ mice perhaps explaining the preponderance of leukemias with mixed lineage expression in both these genotypes (Supplementary Figure 2, Supplemental Digital Content). Leukemias from *Mx*^*T*^*;Ezh2*^*Δ/Δ*^ and *NHD13*^*T*^ exhibited increased *HoxA9* expression compared to WT bone marrow although to a lesser degree in *Mx*^*T*^*;Ezh2*^*Δ/Δ*^ (mean fold change 1.9 vs 4.3 in *NHD13*^*T*^, p = 0.027). There was no further increase in *HoxA9* expression in most leukemias from *NHD13*^*T*^*;Mx*^*T*^*;Ezh2*^*Δ/Δ*^ mice (Fig. 1H).

This study demonstrates a contribution of *Ezh2* loss-of-function to *NHD13*^*T*^-driven MDS and leukemia, however, there was only a mild acceleration of disease onset and similar spectrum of blood cancers. The effect of *Ezh2* deletion in this model may be abrogated for a number of reasons. First, substantial levels of H3K27me3 were maintained in the absence of *Ezh2* expression. From H3K27me3 chromatin immunoprecipitation (CHIP)-sequencing studies, ∼79% of Ezh2 target loci had compensatory methylation mediated by Ezh1.^19^ Functionally, Ezh1 compensation attenuated the hematologic phenotype caused by complete PRC2 dysfunction as has been elegantly demonstrated in *Ezh2* knockout vs *Eed* knockout mice^16^ and *Ezh1/Ezh2* double knockout mice.^19^ Given that Ezh2 and Ezh1 are the only known methyltransferases capable of H3K27 trimethylation in mammals,^6^ we presume the residual H3K27me3 demonstrated in the absence of Ezh2 was mediated by Ezh1 with consequent functional compensation for *Ezh2* loss in our model, which we believe may have attenuated its phenotypic effects. Nonetheless, *EZH1* mutations are not seen in myeloid malignancy and thus these compensatory mechanisms are also likely to be active in human MDS. Second, Ezh2 protein levels were markedly reduced in *NHD13*^*T*^ mice despite RNA expression showing a trendwise increase. Given the maintained H3K27me3 levels, it is likely that Ezh1 compensation was limiting effects of loss of Ezh2. Third, epigenetic mutations, including those in *EZH2*, do not occur late in MDS pathogenesis.^3^ In our study, *Ezh2* deletion was induced relatively late in disease pathogenesis after MDS features such as thrombocytopenia and leucopenia were already present in *NHD13*^*T*^. The timing of deletion may have also abrogated the influence of *Ezh2* deletion in altering the course of *NHD13*^*T*^ disease and is an inherent limitation of the transgenic *NHD13* model. Finally, *EZH2* loss has been previously shown to upregulate *HOX* clusters, including *HOXA* genes, via reduction of H3K27me3 repression in human MDS.^12,23^ The potential overlap in mechanisms of transformation with *NHD13*^*T*^ and *Ezh2* loss through *Hox* gene dysregulation and lack of further de-repression of HoxA9 in *NHD13*^*T*^*;Mx*^*T*^*;Ezh2*^*Δ/Δ*^ may explain the lack of *in vivo* synergy.

*Mx*^*T*^*;Ezh2*^*Δ/Δ*^ mice in this model exhibited a highly penetrant leukemia with most mice succumbing to acute leukemia during the observation period with a median survival of 273 days. Our findings are most similar to the report by Simon et al where all mice exclusively developed T-ALL after a latency of approximately 10 months.^20^ In contrast, in other models using a tamoxifen-inducible Cre-ERT, *Ezh2*-deleted mice developed features of MDS, MPN and MDS/MPN^17–19^ although in 2 of these reports, had similar overall survival to wild-type controls over a period of 300 days’ observation.^17,19^ Two studies reported effects of *Ezh2* deletion in combination with other mutations seen in myeloid malignancy, *Runx1* mutation and *Tet2* knockdown and demonstrated greater acceleration of hematological malignancy from *Ezh2* deletion compared to the *NHD13*^*T*^ background. In combination with *Runx1* mutation, *Ezh2* loss led to a median overall survival of 262 days compared to ‘not reached’ in either mutation alone over 10 months’ observation.^18^ Similarly on a *Tet2* knockdown background, deletion of *Ezh2* accelerated death (median ∼180 days) compared with a ∼300 day median overall survival in mice with either single mutation.^17^ Altogether, these studies highlight diverse, context-dependent outcomes of *Ezh2* deletion in mouse models.

In conclusion, this study describes a model of *EZH2* deletion in MDS, adding to existing literature on the cooperation of *Ezh2* with other genetic aberrations in MDS pathogenesis.^17,18^ Our findings suggest *Ezh2* loss may have limited effects in the *NHD13*^*T*^ given active *Ezh1* compensation and overlapping mechanisms of transformation and highlights caveats in pre-clinical modeling of disease states.

## Supplementary Material

Supplemental Digital Content
