# Supplementary material for: Attenuated Acceleration to Leukemia after Ezh2 Loss in Nup98-HoxD13 (NHD13) Myelodysplastic Syndrome
Source: Hemasphere. 2019 Jul 22;3(4):e277. doi: 10.1097/HS9.0000000000000277 (PMC6745923; doi:10.1097/HS9.0000000000000277)
Supplement: Supplemental Digital Content [file hs9-3-e277-s001.docx]

**Supplemental material**

**Attenuated acceleration to leukemia after EZH2 loss in NHD13 myelodysplastic syndrome, Ling et al.**

**Methods**

**Mice**

All experiments were approved by the respective institutions’ Animal Ethics committees. *NHD13^T^* (ref. ^1^) and *Ezh2^fl/fl^* (ref. ^2^) mice bred on C57BL/6 backgrounds were used. For conditional deletion of *Ezh2*, *Ezh2^fl/fl^* mice were crossed with Mx1-Cre transgenic mice (*Mx^T^*). These strains were bred together to generate four phenotypes (genotypes): wild-type (WT) (*Ezh2^fl/fl^* or *Mx^T^*), *Ezh2*-deleted only (*Mx^T^;Ezh2^Δ/Δ^*), *NHD13^T^* only (*NHD13^T^;Ezh2^fl/fl^* or *NHD13^T^;Mx^T^*) and *NHD13^T^* with *Ezh2* deletion (*NHD13^T^;Mx^T^;Ezh2^Δ/Δ^*). *Ezh2* deletion was induced by intraperitoneal injection of polyinosinic:polycytidylic acid (poly(I:C)) (high molecular weight) (Invivogen Cat#tlrl-pic-5). Poly(I:C) was reconstituted at a concentration of 1 mg/mL and administered at a dose of 6 x 100 μL injections over 2 weeks (Day 1, 3, 5, 8, 10, 12). *Ezh2* deletion was induced in young 6-12 week old mice for long-term survival analyses and in 6 month old mice for hematopoietic stem and progenitor cell (HSPC) analyses. For HSPC analyses, bone marrow was harvested from mice 6-10 weeks after the start of poly(I:C) administration. Mice used for WT whole bone-marrow and kit-enriched bone marrow controls for Western blot and quantitative polymerase chain reaction (qPCR) were C57BL/6J adult mice.

Peripheral blood was obtained by submandibular bleeds into EDTA. Full blood counts were obtained on a Hemavet instrument (Drew Scientific). At death, bone marrow, spleen and if present, thymus from mice were harvested and processed to single-cell suspensions by routine methods. Cells underwent red cell lysis in lysis buffer (BD Pharm Lyse solution) for 5 minutes and washed in phosphate buffered saline (PBS) + 2% fetal calf serum (FCS) before further processing. Bone marrow samples used for Western blot and qPCR are listed in Supplementary Table 4.

**Western blot**

Protein expression was assessed by Western blot using routine methods. 2 x 10^6^ cells were washed twice with PBS and lysed in 200 μL of lysis buffer (30 mM Tris HCl pH 6.8, 4% sodium dodecyl sulfate (SDS), 5% glycerol, 2.5% β-mercaptoethanol and 0.2% bromophenol blue). 20 μL of lysate was resolved on pre-cast 4-20% polyacrylamide gels (Biorad) at 80V for 20 mins, followed by 120V for ~40 mins and transferred to polyvinylidene difluoride (PVDF) membranes at 100V for 1 hour. After blocking in 5% skim milk in tris-buffered saline with Tween (TBST) (20 mM Tris, 0.15 mM NaCl, Tween 0.1%, pH 7.5), the membrane was probed with the following primary antibodies: Ezh2 (Cell Signaling Technology, Cat#4905) or H3K27me3 (Diagenode, Cat#C15410195) at 1:1000 in TBST / 5% skim milk overnight at 4°C or Actin antibody (BD Biosciences, Cat# 612656) at 1:5000 in TBST / 5% skim milk for 1 hour as a loading control, followed by anti-rabbit or -mouse secondary antibodies. Immunodetection was performed using Clarity™ Western ECL Substrate (BioRad, Cat# 1705061).

**Flow cytometry**

The following flow cytometry antibodies from BD Biosciences were used to determine immunophenotype of mouse leukemias: c-kit PerCP Cy5.5 (1:100), CD3 PeCy7 (1:500), B220 FITC (1:250), CD19 APC Cy7 (1:500), Gr1 PE (1:1000) and Mac1 APC (1:250). For HSPC analyses, the following antibodies, also from BD Biosciences were used: lineage biotin (CD3 (1:500), CD4 (1:1000), CD8 (1:1000), B220 (1:500), Gr1 (1:1000), Mac1 (1:500) and Ter119 (1.500)) with streptavidin PerCP Cy5.5 (1:500) secondary antibody, Sca1 PeCy7 (1:100), ckit APC (1:100), CD48 APCCy7 (1:100) and CD150 PE (1:100). Flow cytometry analyses were performed on the LSRII instrument (BD).

**PCR and qPCR**

Total RNA was isolated using Trizol (Invitrogen) or the MicroRNAeasy kit (Qiagen) according to manufacturer instructions. RNA from Trizol samples underwent dsDNase treatment (Thermofisher) to remove contaminating genomic DNA. RNA was reverse transcribed into complementary DNA (cDNA) using the Maxima H Minus First Strand cDNA synthesis kit (Thermofisher) using random hexamer primers.

To confirm maintained *Nup98:HoxD13* expression in end-leukaemic samples, PCR was performed on cDNA from selected samples across genotypes with the following primers: NUP98 (forward), 5′-TGGAGGGCCTCTTGGTACAGG-3′; HoxD13-L1 (reverse), 5′-GGCTTCTAAGCTGTCTGTGGCC-3′. The amplification protocol was: 94°C for 3 minutes, followed by 35 cycles of 94°C for 30 seconds, 62°C for 30 seconds, and 72°C for 1 minute, followed by a final extension phase at 72°C for 12 minutes.

For gene expression quantitation, real-time qPCR was performed using the ABI Viia 7 instrument using SYBR Green PCR Master Mix (Thermofisher). Primer sequences are listed below:

| **Gene** | **Forward primer 5’-3’** | **Reverse primer 5’-3’** |
| --- | --- | --- |
| *Ezh2* | CAACCCGAAAGGGCAACAAA | TCACCAGTCTGGATAGCCCT |
| *Ezh1* | TCAAACGGCTCCAGGCAAATA | GGACACGAAGTTTCTTCCACTC |
| *HoxA9* | CTGTCCCACGCTTGACACTC | CTCCGCCGCTCTCATTCTC |
| *Sfrp1* | TCTAAGCCCCAAGGTACAACC | GCTTGCACAGAGATGTTCAATG |
| *CD109* | TCCCGCTTTCTGGTGACAG | ACCTGAGCCTTTACAAGGACC |
| *Gapdh* | AGGTCGGTGTGAACGGATTTG | TGTAGACCATGTAGTTGAGGTCA |
| *Actin* | GACGATATCGCTGCGCTGGT | CCACGATGGAGGGGAATA |

Relative quantification of expression was performed using a standard curve covering cDNA dilutions that encompassed the concentrations of the unknown samples and normalized to Actin and GAPDH. Expression levels were expressed as a fold change compared with WT whole bone marrow.

**Statistical analysis**

Groups were compared using analysis of variance (ANOVA) followed by Tukey’s multiple testing or unpaired t-testing. Leukemia-free survival was determined from the start date of poly(I:C) administration. Mice deemed to have died due to poly(I:C) toxicity were specifically excluded from survival curves. Mice alive ~14 months after poly(I:C) were censored. Survival curves were compared using the Log-rank test. Pairwise Fisher exact testing was used to compare proportions of leukemic subtypes across genotypes. A p-value of <0.05 was considered significant. Statistics were performed using Prism7.

**Supplementary Tables**

**Supplementary Table 1. Bone marrow leukemia samples used for Western blot and qPCR**

| **Sample#** | **Genotype** | **Age at Poly(I:C) (days)** | **Age at death (days)** | **Cause of death** | **Ckit%** |
| --- | --- | --- | --- | --- | --- |
| 935 | *EZH2^Δ/Δ^* | 48 | 318 | MPAL (B/T) | 18.5 |
| 936 | *EZH2^Δ/Δ^* | 48 | 254 | MPAL (B/Myeloid) | 13.6 |
| 938 | *EZH2^Δ/Δ^* | 52 | 305 | T-ALL | 12.4 |
| 877 | *NHD13^T^* | 100 | 398 | T-ALL | 52.5 |
| 929 | *NHD13^T^* | 51 | 222 | T-ALL | 92.5 |
| 932 | *NHD13^T^* | 48 | 226 | AML | 40 |
| 880 | *NHD13^T^;EZH2^Δ/Δ^* | 100 | 294 | T-ALL | 10 |
| 910 | *NHD13^T^;EZH2^Δ/Δ^* | 66 | 347 | AML | 14.5 |
| 933 | *NHD13^T^;EZH2^Δ/Δ^* | 48 | 311 | T-ALL | 49.5 |

AML, acute myeloid leukemia; ALL, acute lymphoid leukemia; MPAL, mixed phenotype acute leukemia.

**Supplementary Table 2. Hematologic malignancy in *NHD13^T^* mice**

| ID | Age, days | Time from Poly(I:C), days | Spleen weight, g | Hb, g/L | MCV, fL | WCC, x10^9^/L | Plts, x10^9^/L | **Diagnosis** |
| --- | --- | --- | --- | --- | --- | --- | --- | --- |
| 920 | 138 | 77 | N/A | 114 | 49.3 | 13.1 | 215 | - |
| 889 | 218 | 130 | 0.99 | 78 | 76.4 | 20 | 277 | AML |
| 929 | 222 | 171 | 1.84 | 62 | 51.3 | ++++ | 47 | T-ALL |
| 932 | 226 | 178 | 1.31 | 52 | 53.1 | ++++ | 92 | AML |
| 891 | 238 | 150 | N/A | 123 | 46.6 | 10.3 | 258 | - |
| 985 | 249 | 184 | N/A | 139 | 55.8 | 1.84 | 536 | - |
| 908 | 253 | 187 | 0.5 | 56 | 73.7 | 13.5 | 100 | B-ALL |
| 885 | 280 | 185 | N/A | 111 | 52.7 | 3.1 | 742 | - |
| 900 | 303 | 224 | N/A | 104 | 59.4 | 57.2 | 105 | - |
| 901 | 349 | 270 | 0.53 | 73 | 48.8 | 34.2 | 157 | B-ALL |
| 948 | 353 | 305 | N/A | 94 | 62.5 | 12.2 | 464 | - |
| 951 | 367 | 320 | 1.94 | 65 | 70.4 | 160.7 | 366 | AML |
| 878 | 370 | 270 | 1.25 | 42 | 70.7 | 36.5 | 65 | AML |
| 877 | 398 | 298 | N/A | 56 | 50.4 | 17 | 134 | T-ALL |
| 947 | 409 | 361 | 0.61 | 57 | 54.4 | 26 | 335 | B-ALL |
| 953 | 409 | 362 | 1.18 | 34 | 49.7 | 12.7 | 117 | MPAL (T/myeloid) |
| 886 | 436 | 341 | 1.03 | 115 | 75.9 | ++++ | 1035 | AML |
| 884 | 440 | 345 | 1.19 | 42 | 67.9 | 13.1 | 268 | T-ALL |

Hb, hemoglobin; MCV, mean corpuscular volume; WCC, white cell count; Plts, platelets; AML, acute myeloid leukemia; ALL, acute lymphoid leukemia; MPAL, mixed phenotype acute leukemia. N/A = not attained, ‘-‘ no or limited necropsy performed to obtain a diagnosis.

**Supplementary Table 3. Hematologic malignancy in *Ezh2^Δ/Δ^* mice**

| ID | Age, days | Time from PolyIC, days | Spleen weight, g | Hb, g/L | MCV, fL | WCC, x10^9^/L | Plts, x10^9^/L | **Diagnosis** |
| --- | --- | --- | --- | --- | --- | --- | --- | --- |
| 909 | 239 | 173 | 0.74 | 93 | 50.7 | 4.2 | 232 | T-ALL |
| 936 | 254 | 206 | 0.13 | 130 | 46.9 | 3.4 | 511 | MPAL (B/myeloid) |
| 902 | 275 | 196 | 0.45 | 127 | 44.7 | 14.3 | 600 | MPAL (B/T) |
| 1015 | 277 | 205 | N/A | 138 | 42.7 | 5.46 | 874 | - |
| 991 | 279 | 214 | 0.5 | 123 | 54.5 | 12.8 | 546 | AML |
| 938 | 305 | 253 | N/A | 94 | 73.5 | 29.1 | 530 | T-ALL |
| 944 | 305 | 253 | N/A | 138 | 59.4 | 18.3 | 507 | - |
| 1019 | 306 | 240 | 1.09 | 98 | 56.2 | 8.6 | 512 | T-ALL |
| 935 | 318 | 270 | 1.58 | 58 | 85.6 | 74.7 | 395 | MPAL (B/T) |
| 983 | 346 | 262 | 1.01 | 104 | 45.4 | 12.9 | 274 | T-ALL |
| 1022 | 361 | 300 | 0.5 | 42 | 51.8 | 20.3 | 632 | - |
| 990 | 362 | 297 |  | 88 | 50.8 | 41.3 | 663 | MPAL (B/T) |
| 905 | 366 | 300 | N/A | 93 | 50.4 | 5.18 | 920 | - |
| 879 | 383 | 283 | N/A | 102 | 42.4 | 1.5 | 1177 | - |
| 1018 | 400 | 334 | N/A | 144 | 46.6 | 2.28 | 437 | - |
| 988 | 409 | 344 | 0.55 | 32 | 60.2 | 18.7 | 843 | - |
| 937 | 412 | 364 | 1.19 | 92 | 56.6 | 32 | 552 | MPAL (B/T) |
| 1017 | 418 | 352 | 1.6 | 85 | 49.9 | 14.3 | 610 | - |

Hb, hemoglobin; MCV, mean corpuscular volume; WCC, white cell count; Plts, platelets; AML, acute myeloid leukemia; ALL, acute lymphoid leukemia; MPAL, mixed phenotype acute leukemia. N/A = not attained, ‘-‘ no or limited necropsy performed to obtain a diagnosis.

**Supplementary Table 4. Hematologic malignancy in *NHD13^T^;Ezh2^Δ/Δ^* mice**

| ID | Age, days | Time from PolyIC, days | Spleen weight, g | Hb, g/L | MCV, fL | WCC, x10^9^/L | Plts, x10^9^/L | **Diagnosis** |
| --- | --- | --- | --- | --- | --- | --- | --- | --- |
| 993 | 208 | 143 | 1.53 | 32 | 58.9 | 8.8 | 310 | T-ALL, AML |
| 888 | 244 | 156 | 1.34 | 42 | 57.8 | 26.8 | 96 | AML |
| 987 | 259 | 194 | 0.15 | 79 | 54.2 | 1.42 | 502 | T-ALL |
| 897 | 266 | 187 | N/A | 88 | 59.2 | 5.3 | 320 | MDS |
| 890 | 272 | 184 | 1.43 | 59 | 77.7 | ++++ | 24 | Lineage negative leukaemia |
| 934 | 284 | 236 | 0.13 | 30 | 58.4 | 1.24 | 1104 | MPAL (T/myeloid) |
| 880 | 294 | 194 | 1.4 | 55 | 61 | ++++ | 334 | T-ALL |
| 1021 | 299 | 238 | 0.92 | 79 | 82.8 | ++++ | 382 | T-ALL |
| 933 | 311 | 263 | 2.13 | 190 | 86 | ++++ | 1342 | T-ALL |
| 896 | 325 | 246 | 0.25 | 124 | 52.8 | 8.4 | 140 | T-ALL |
| 962 | 328 | 283 | 0.27 | 28 | 68.5 | 4.86 | 484 | T-ALL |
| 1025 | 329 | 268 | N/A | 118 | 52 | 8.8 | 326 | - |
| 1028 | 340 | 279 | N/A | 144 | 49.6 | 2.26 | 362 | - |
| 910 | 347 | 281 | 1.2 | 70 | 73.8 | 74.5 | 453 | AML |
| 903 | 349 | 270 | 1.58 | 42 | 68.3 | 75.1 | 51 | Lineage negative leukaemia |

Hb, hemoglobin; MCV, mean corpuscular volume; WCC, white cell count; Plts, platelets; AML, acute myeloid leukemia; ALL, acute lymphoid leukemia; MPAL, mixed phenotype acute leukemia. N/A = not attained, ‘-‘ no or limited necropsy performed to obtain a diagnosis.

**Supplementary Table 5. P-value results of pairwise Fisher exact testing comparing proportions of leukemia subtypes across genotypes***.*

|  | T-ALL | B-ALL | AML | AL - other | MDS |
| --- | --- | --- | --- | --- | --- |
| *Ezh2^Δ/Δ^* vs *NHD13^T^* | 0.6517 | 0.2208 | 0.1619 | 0.0557 | >0.9999 |
| *Ezh2^Δ/Δ^* vs *NHD13^T^;Ezh2^Δ/Δ^* | >0.9999 | >0.9999 | >0.9999 | 0.4173 | >0.9999 |
| *NHD13^T^* vs *NHD13^T^;Ezh2^Δ/Δ^* | 0.4110 | 0.0957 | 0.2016 | 0.3217 | >0.9999 |

**Supplementary Figures**


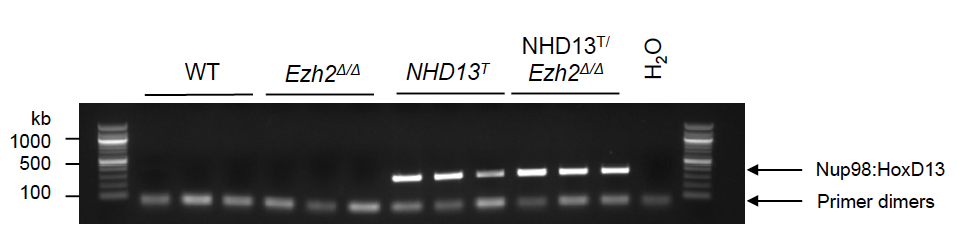


**Supplementary Figure 1.** PCR for the *Nup98:HoxD13* fusion gene from cDNA from selected leukemic samples. Sample identities are listed in Supplementary Table 1 and are loaded in the order listed.

**Supplementary Figure 2. Hematopoietic stem cell proportions in non-leukaemic mice. A)** Lineage negative, Sca1^+^, c-kit^+^ (LSK) fraction as a percentage of bone marrow cells. B) LSK sub-populations defined by CD48 and CD150 staining. LT-HSCs: CD48^-^, CD150^+^; ST-HSCs: CD48^-^, CD150^-^; MPP2: CD48^+^, CD150^+^; MPP3/4: CD48^+^, CD150^-^. C) Statistics for individual LSK subpopulations. Statistics shown are the result of ANOVA comparisons, followed by Tukey’s multiple comparison’s test. *p<0.05, **p<0.01, ***p<0.001, ****p<0.0001.

**Supplemental References**

1. Lin Y-W, Slape C, Zhang Z, Aplan PD. NUP98-HOXD13 transgenic mice develop a highly penetrant, severe myelodysplastic syndrome that progresses to acute leukemia. *Blood.* 2005;106(1):287-295.

2. Su I-h, Basavaraj A, Krutchinsky AN, et al. Ezh2 controls B cell development through histone H3 methylation and Igh rearrangement. *Nature immunology.* 2003;4(2):124.
